# Enhancing the solubility of puerarin via melt sonocrystallization

**DOI:** 10.1371/journal.pone.0355646

**Published:** 2026-08-07

**Authors:** Yaxiang Gong, Jiafu Wen, Wenqi Liu

**Affiliations:** 1 Department of Pharmacy, Linyi People’s Hospital, Linyi, Shandong, China; 2 Third Ward of Hand and Foot Surgery Department, Linyi People’s Hospital, Linyi, Shandong, China; China Academy of Chinese Medical Sciences Institute of Chinese Materia Medica, CHINA

## Abstract

In this work, in order to improve the low solubility and poor oral bioavailability of puerarin, we first applied melt sonocrystallization (MSC) technology to systematically investigate the effects on its solubility and crystallographic properties. We applied MSC to treat puerarin, evaluated its solubilization effect using UV spectroscopy, and characterized it by dissolution rate, optical microscope, powder X-ray diffraction (PXRD), differential scanning calorimetry (DSC), Thermogravimetric analysis (TGA) and Fourier transform infrared spectroscopy (FTIR). Following MSC treatment, puerarin exhibited higher solubility and faster dissolution, the product displayed reduced particle size and a tendency to agglomerate. The results of PXRD, DSC, FTIR and TGA showed that the crystal form of MSC treated puerarin transformed into puerarin monohydrate, with a decrease in crystallinity and partial amorphization. The reduction in particle size and amorphization may be the driving forces of the increase in solubility and dissolution. In summary, MSC can significantly improve the solubility and dissolution of puerarin, providing new ideas for enhancing its bioavailability and improving its oral formulations.

## Introduction

Oral administration remains the most common and convenient route in clinical practice; however, a large amount of new molecular entities are abandoned because of poor aqueous solubility, which leads to incomplete dissolution in the gastrointestinal fluids and, consequently, inadequate systemic exposure [1–5]. Therefore, how to improve the apparent solubility and dissolution rate of drugs through pharmaceutical methods while maintaining the stability of their chemical properties has become a key scientific problem that urgently needs to be solved in the field of pharmacy.

Traditional solubilization strategies such as reducing particle size, preparing cocrystal, preparing solid dispersions or cyclodextrin inclusion complexes can improve the solubility and dissolution of drugs to a certain extent, but they generally have drawbacks such as complex processes, residual organic solvents, or poor stability [6–11]. In recent years, MSC has provided new ideas for the research of insoluble drugs due to its ability to induce nucleation, refine crystals, and regulate crystal habits through cavitation effect under mild conditions [12,13]. MSC developed on this basis couples drug melting, ultrasonic dispersion, and rapid solidification, synergistically improving the solubility and dissolution rate. For example, Vaibhavkumar et al. improved the physicochemical properties of rosiglitazone through MSC, and increased its solubility and dissolution [14]; M Manish et al. improved the physicochemical properties of ibuprofen through melt sonocrystallization technology, resulting in improved compressibility [15]. MSC could effectively regulate the crystal form and particle size of active pharmaceutical ingredients by precisely controlling the ultrasound frequency, intensity, and duration. This process can transform drugs that were originally difficult to dissolve in water into uniform, stable, and controllable crystals, thereby increasing the solubility, significantly improving its dissolution rate and bioavailability, reducing the use of additional excipients, and lowering potential adverse reactions.

Puerarin is a small molecule drug extracted from the plant pueraria lobata, which belongs to isoflavone compounds [16–20]. It has pharmacological effects such as coronary artery dilation, antioxidant, anti-inflammatory, etc [21–25]. Therefore, it could be used in clinical treatment of cardiovascular and cerebrovascular diseases and diabetes [26–29]. However, as a biopharmaceutics classification system (BCS) Class IV drug, puerarin displays poor equilibrium solubility in water, leading to insufficient oral bioavailability and constituting a key problem that restricts its formulation development and clinical efficacy [30–33]. Therefore, selecting an appropriate strategy to overcome the physicochemical limitations of puerarin and break through its solubility barrier is of great significance for further improving its oral bioavailability and promoting the development of oral formulations. Several strategies have been employed to improve the solubility of puerarin specifically, including nanoparticle formulation, solid dispersions, and cocrystals. However, these methods often suffer from complex preparation processes, residual organic solvents, or poor long-term stability. In contrast, melt sonocrystallization (MSC) offers a solvent-free, single-step approach that reduces particle size. To date, MSC has not been applied to puerarin.

In view of this, we selected puerarin as the model drug to systematically investigate the influence of MSC on its crystallographic properties and solubility, aiming to provide an efficient and green reference for the solubilization of poorly soluble drugs. We prepared solubilized puerarin with higher solubility and dissolution rate via melt sonocrystallization and conducted systematic characterization-morphological analysis, powder X-ray diffraction, thermogravimetric analysis and infrared spectroscopy-laying a solid foundation for further exploration of puerarin formulation potential and optimization of its clinical application, while also offering insights for enhancing the solubility of other poorly soluble drugs.

## Materials and methods

### Materials

Puerarin (≥98%) was purchased from Solarbio Science & Technology Co., Ltd. (Beijing, China). Potassium bromide (≥99%) was obtained from Bide Pharma Co., Ltd. (Shanghai, China). Ultrapure water (18.2 MΩ·cm) was produced with a Milli-Q system (Millipore, USA).

### Melt sonocrystallization of puerarin

Exactly 400mg of puerarin was weighed using an analytical balance (Shanghai Liangping Instrument Co., Ltd., China) and heated to 206°C until molten. The molten material was immediately poured into 8 mL of ice-cold deionised water under continuous stirring, contained in a cylindrical glass vessel with about 20 mm diamater. The suspension was immediately transferred to an ice bath and sonicated with a probe-type ultrasonic disruptor (Ningbo Xinzhi Biotechnology Co., Ltd., China) using 5-s pulses followed by 5-s rest intervals for a total processing time of 20 min, the operation parameters were as follow: ultrasonic frequency of 20 kHz, output power of 300 W, probe diameter of 6 mm, and probe immersion depth of about 10 mm below the liquid surface, ensuring complete immersion without contact with the vessel bottom. During the ultrasound process, the suspension was consistently cooled in an ice bath. The resultant dispersion was filtered, and the collected solid was dried under vacuum for 12 h to yield the melt-sonocrystallized product (MSC-puerarin).

### Morphological analysis

A small quantity of raw puerarin was dusted onto a glass slide, dispersed in a drop of liquid paraffin, and examined under a optical microscope (Olympus, Japan). After MSC treatment, an aliquot of the still-wet puerarin suspension was similarly transferred onto a slide and inspected.

### Solubility determination

Preliminary experiments were conducted to establish the time required to reach thermodynamic equilibrium. Excess MSC-puerarin (approximately 50 mg) was added to 5 mL of deionized water in sealed vials and shaken at 25°C (150 rpm). Aliquots were withdrawn at 6, 12, 24, and 48 hours, filtered (0.22 µm), and analyzed by UV spectroscopy at 250 nm. Equilibrium was confirmed when consecutive measurements differed by less than 5% (typically achieved by 12 hours). Based on the equilibration study, excess amounts of untreated puerarin or MSC-puerarin were introduced into 5 mL of deionized water. For each experimental run, one aliquot of raw puerarin and one aliquot of MSC-puerarin were measured under identical conditions. The suspensions were equilibrated at 25, 30, 35, 40, or 45°C under shaking for 12h. After filtration (0.22 µm), the filtrate was analysed at 250 nm using UV spectroscopy mode, this process was operated using a SpectraMax M5 microplate reader (Molecular Devices, USA) and each experiment was performed in triplicate (n = 5).

### Dissolution rate

Powder dissolution was assessed using an RC-806 dissolution tester (Tianda Tianfa Technology Co., Ltd., China). Samples equivalent to 20 mg of puerarin or MSC-puerarin were dispersed in 100 mL deionized water maintained at 25°C or 37.0°C with paddle speed of 50 rpm. Aliquots (2 mL) were withdrawn at 0.17(10 min), 0.5, 1, 2, 4, 7, 10, 12, and 24 h, filtered (0.22 µm), diluted appropriately, and assayed using UV spectroscopy mode of a SpectraMax M5 microplate reader (Molecular Devices, USA) and each experiment was performed in triplicate (n = 5).

### Differential scanning calorimetry (DSC)

Samples of untreated puerarin or MSC-puerarin were analysed by Differential scanning calorimetry. Approximately 3–5 mg of puerarin and MSC-puerarin in an open aluminum crucible. The heating rate was 10°C min-1 and the measurement range was 30–220°C. The data was analyzed using NETZSCH Proteus thermal analysis software (Version 4.2).

### Powder X-ray diffraction

Samples of untreated puerarin or MSC-puerarin were analysed on a Bruker D8 Advance diffractometer (Cu Kα, λ = 1.5406 Å, 40 kV, 40 mA). Data were collected between 2θ = 5−30°, the step was 0.05° and the scan speed was 4° min^-1^.

### Fourier transform infrared spectrophotometry (FTIR)

Take potassium bromide crystals and dry them at 60°C for 10 hours. Take an excess of puerarin raw material and MSC-treated puerarin, mix them with potassium bromide in a mass ratio of 1:50, press them into tablets, scan them in the range of 4000-400cm^-1^, and perform infrared spectroscopy analysis using a Nicolet iS5 FT-IR spectrometer (Thermo Fisher Scientific, USA).

### Karl Fischer moisture determination

After vacuum dried at 25°C for 24 h, the water contents of MSC-puerarin were measured by a V20 Karl-fischer moisture meter (Mettler Toledo, Switzerland). 100 mg MSC-puerarin were carefully weighed and titrated by anhydrous methanol.

### Thermogravimetric analysis (TGA)

Samples of MSC-puerarin were analysed by TGA 4000 thermogravimetric analyzer (USA). Approximately 3–5 mg of MSC-puerarin was placed in the perforated aluminum pan system for test. The heating rate was 10°C min^-1^ and the measurement range was 30–195°C.

### Statistical analysis

All quantitative experiments were performed in independent replicates (n = 5). Data are presented as mean ± standard deviation (SD). Normality of data distribution was assessed using the Kolmogorov- Smirnov test and the Shapiro-Wilk test. Comparisons between raw puerarin and MSC-puerarin were performed using paired samples t-test for normally distributed data. Exact p-values are reported for each comparison, and the significance threshold was set at p < 0.05. Error bars in all figures represent SD. Statistical analyses were conducted using SPSSAU(an online statistical analysis platform, which implements identical statistical algorithms to IBM SPSS Statistics, https://spssau.com/?107000000).

## Results and discussion

### Morphological analysis

In this work, MSC-puerarin solid powder was prepared. Although puerarin exhibits poor aqueous solubility at ambient temperature, its solubility increases markedly with temperature. According to Pang et al., concentrated puerarin solutions form a macroscopically homogeneous gel upon cooling to room temperature; the resulting gel shows no flow under gravity when inverted [34]. To prevent gel formation and to favor precipitation of the solid, the ultrasound procedure was performed under an ice bath, rapidly lowering the temperature and thus the solubility of puerarin. Simultaneously, ultrasonic disruption prevented the establishment of a gel network, ensuring that only solid particles were recovered.

**Fig 1:**
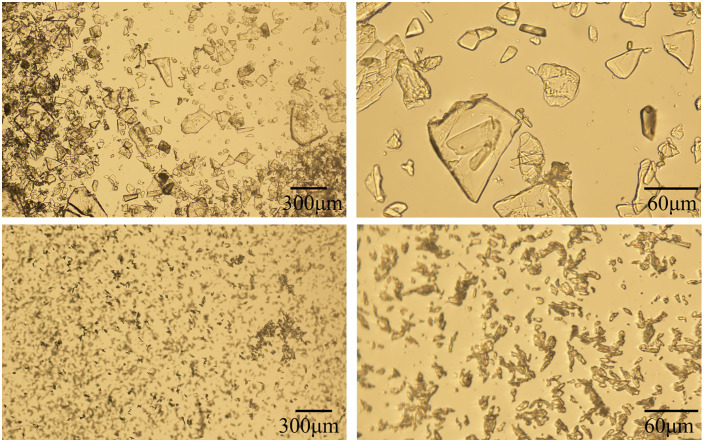
Morphological analysis under optical microscopy. **a,** The morphology of raw puerarin under 10 × 10 magnification. **b,** raw puerarin under 40 × 10 magnification. **c,** MSC-puerarin under 10 × 10 magnification.**d,** MSC-puerarin under 40 × 10 magnification. Raw puerarin appears as distinct, plate-like crystals, whereas the MSC product tends to agglomerate and consists predominantly of dot-like or short rod-shaped particles with substantially smaller dimensions.

Optical microscopy (Fig 1) reveals pronounced morphological differences between the raw and MSC treated materials. At 10 × 10 magnification, raw puerarin appears as distinct, plate-like crystals, whereas the MSC product tends to agglomerate. At 40 × 10, the processed material consists predominantly of dot-like or short rod-shaped particles whose dimensions are substantially smaller than those of the original crystals.

### Solubility

**Fig 2:**
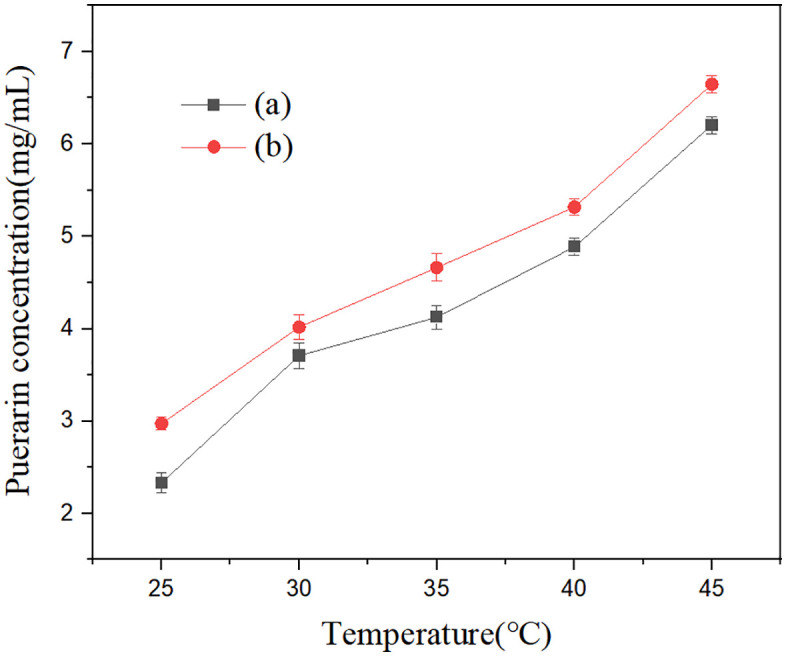
The solubility-temperature profiles of puerarin. **a,** raw puerarin. **b,** MSC-puerarin. Data are presented as mean ± SD (n = 5). Statistical comparisons were performed using paired samples t-test. Exact p-values: 25°C, p < 0.001; 30°C, p = 0.016; 35°C, p = 0.004; 40°C, p = 0.002; 45°C, p = 0.001. *p < 0.05 vs. raw puerarin.

As showed in the solubility-temperature profiles (Fig 2), over the entire 25–45°C range, the solubility of MSC-puerarin was markedly higher than that of the untreated drug, with the most pronounced improvements observed at 25 and 35°C. At 25°C, the solubility of MSC-puerarin was 2.97 ± 0.07 mg/mL, representing a 27.5% increase compared to raw puerarin (2.33 ± 0.11 mg/mL) (p < 0.001). At 30°C, the solubility of MSC-puerarin was 4.02 ± 0.14 mg/mL, representing a 8.4% increase compared to raw puerarin (3.71 ± 0.14 mg/mL) (p = 0.016). At 35°C, the solubility of MSC-puerarin was 4.66 ± 0.15 mg/mL, representing a 13.1% increase compared to raw puerarin (4.12 ± 0.13 mg/mL) (p = 0.004). At 40°C, the solubility of MSC-puerarin was 5.32 ± 0.08 mg/mL, representing a 8.8% increase compared to raw puerarin (4.89 ± 0.09 mg/mL) (p = 0.002). At 45°C, the solubility of MSC-puerarin was 6.65 ± 0.09 mg/mL, representing a 7.3% increase compared to raw puerarin (6.20 ± 0.10 mg/mL). These data provide clear evidence that MSC enhances the thermodynamic solubility of puerarin (P < 0.05). Microscopic examination revealed that MSC markedly reduced the particle size of puerarin. The smaller dimensions confer higher specific surface energy and a larger interfacial area upon contact with the dissolution medium, which is believed to be a primary reason for the observed increases in solubility [35,36].

### Dissolution

**Fig 3:**
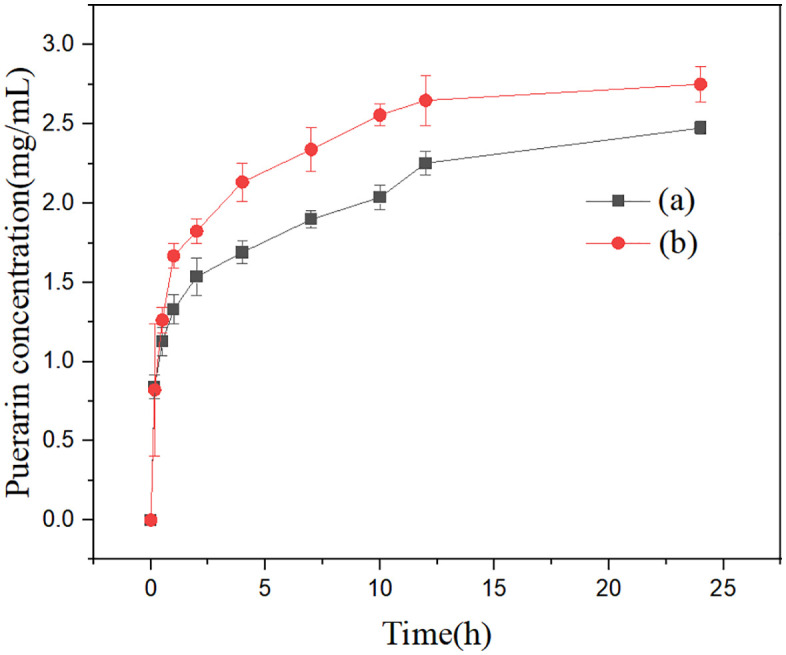
The dissolution profiles of puerarin at 25°C. **a,** raw puerarin. **b,** MSC-puerarin. Data are presented as mean ± SD (n = 5). Statistical comparisons were performed using paired samples t-test. Exact p-values: 0.5h, p = 0.035; 1h, p = 0.01; 2h, p = 0.007; 4h, p = 0.003; 7h, p = 0.002; 10h, p < 0.001; 12h, p = 0.002; 24h, p = 0.009. *p < 0.05 vs. raw puerarin.

**Fig 4:**
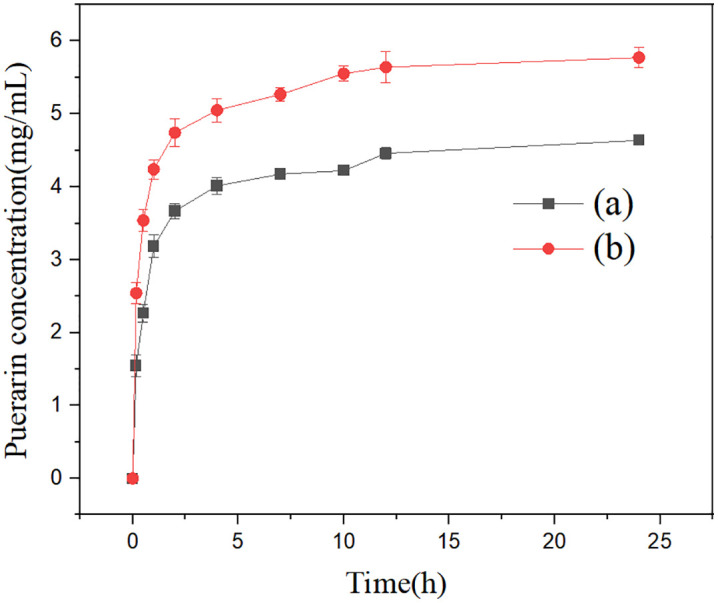
The dissolution profiles of puerarin at 37°C. **a,** raw puerarin. **b,** MSC-puerarin. Data are presented as mean ± SD (n = 5). Statistical comparisons were performed using paired samples t-test. Exact p-values: 0.17h, p = 0.001; 0.5h, p < 0.001; 1h, p = 0.001; 2h, p < 0.001; 4h, p = 0.001; 7h, p < 0.001; 10h, p < 0.001; 12h, p < 0.001; 24h, p < 0.001. *p < 0.05 vs. raw puerarin.

Due to the extremely low aqueous solubility of puerarin, sink-condition dissolution testing would necessitate non-physiological solvent volumes that obscure concentration-dependent precipitation. We therefore utilized supersaturating conditions to better reflect in vivo dissolution behavior. Dissolution profiles (Figs 3 and 4) reveal marked improvements for the MSC product at both 25°C and 37°C. At 25°C, the raw material released 67.7% within 4 h and 90.0% after 12 h, whereas the MSC product reached 77.5% and 96.4%, respectively. The peak concentration increased by approximately 10.0%, and the dissolution rate was notably accelerated. At 37°C, the differences were even more evident: raw puerarin released 89.1% at 4 h and 99.1% at 12 h, while MSC-puerarin achieved 89.4% and 99.8%, respectively. The peak concentration rose by 26.5%, and the most pronounced enhancement in dissolution rate occurred within the first 4 h. Collectively, MSC significantly increases both the extent and the rate of puerarin dissolution (P < 0.05). In the same way that MSC reduces particle size, increases specific surface energy, and thereby elevates puerarin solubility, these same physical changes account for the accelerated dissolution observed after processing.

### Differential scanning calorimetry

**Fig 5:**
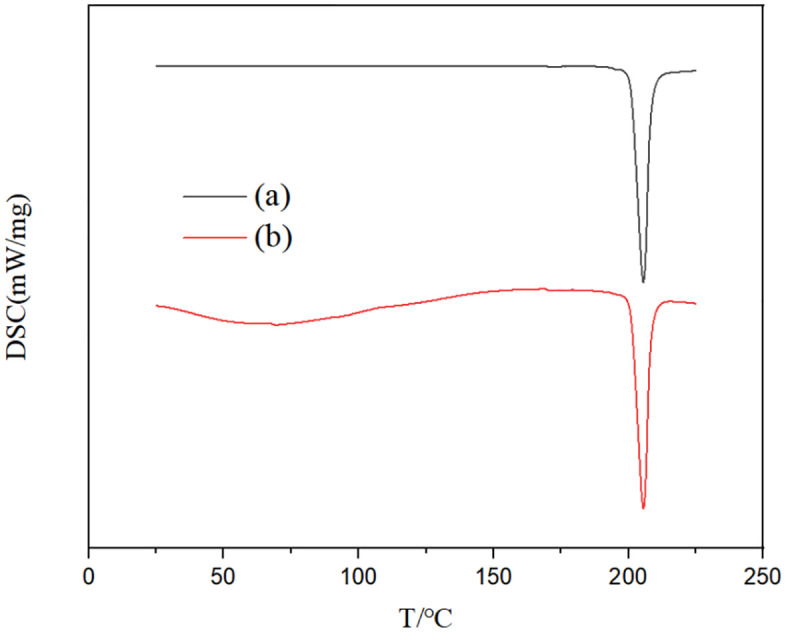
DSC thermograms of puerarin. **a,** DSC of raw puerarin. **b,** DSC of MSC-puerarin. Samples were heated in open aluminum crucibles from 30 to 220°C. MSC-puerarin displays an endothermic peak at 206°C.

As showed in Fig 5, DSC thermograms of MSC-puerarin display an endothermic peak at 206°C, corresponding to the melting of the crystalline phase, and a broad event centred around 50°C. The latter may represent the glass transition of an amorphous fraction, the desolvation of a hydrated form generated during processing, or a superposition of both phenomena.

### Crystal structure analysis

**Fig 6:**
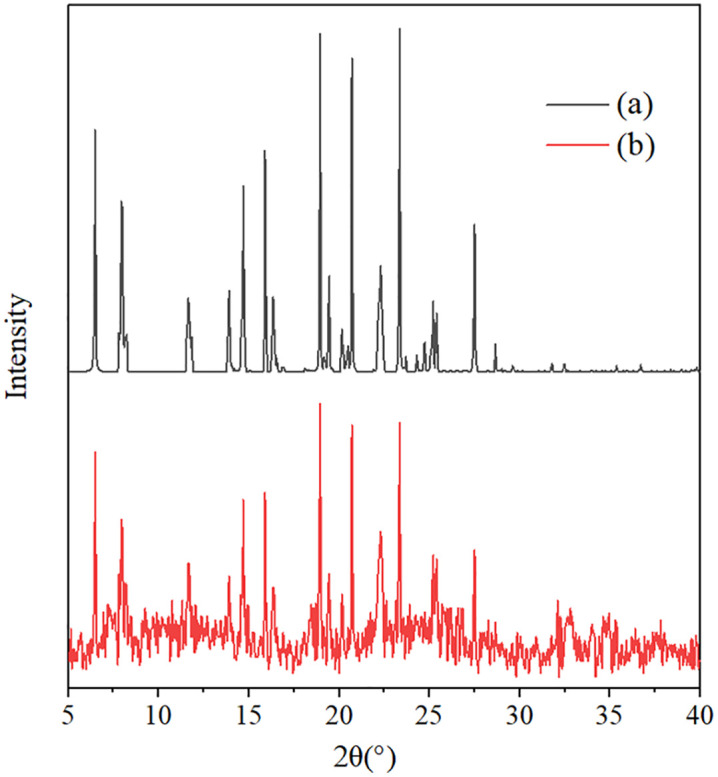
PXRD pattern of puerarin. **a,** XRD pattern of raw puerarin. **b,** XRD pattern of MSC-puerarin. After MSC treatment, peak positions shifte, with decreased peak intensity, elevated baseline, and dispersed pattern, indicating crystal transformation and partial amorphization.

The results of powder X-ray diffraction (PXRD) are shown in Fig 6. The active pharmaceutical ingredient puerarin exhibits sharp and high-intensity characteristic diffraction peaks at 2 θ = 6.7 °, 8.5 °, 11.2 °, 13.7 °, 16.2 °, 16.7 °, 17.4 °, 18.6 °, 19.2 °, 20.2 °, 22.4 °, 23.9 °, 25.4 °, 27.5 °, and has a complete crystal structure. After MSC treatment, the diffraction peak positions of the sample changed to a certain extent, with peak positions becoming 2θ = 6.5 °, 8.0 °, 11.7 °, 13.9 °, 14.7 °, 15.9 °, 16.4 °, 17.1 °, 18.9 °, 19.6 °, 20.8 °, 22.3 °, 23.4 °, 25.2 °, and 27.5 °, indicating that a crystal transformation had occurred. MSC treatment triggered the transformation of puerarin; According to Karl Fischer analysis, the molar ratio of puerarin to water in MSC-puerarin is close to 1:1. We compared the PXRD spectra of MSC-puerarin with reported puerarin monohydrate and found that the peak positions of the two were almost identical [34]. It can be inferred that after treated by MSC, puerarin undergoes crystal transformation into puerarin monohydrate. In addition, the intensity of each characteristic peak significantly decreased, the baseline was raised and showed a dispersed pattern, indicating that the crystallinity of puerarin after treatment decreased, lattice defects and local disorder appeared, and partial amorphization may have occurred.

This phenomenon can be attributed to the cavitation effect during the MSC process: on the one hand, high-temperature melting destroys the regularity of the original lattice; On the other hand, ultrasonic cavitation causes the melted puerarin to freeze some of its amorphous state during the cooling and solidification process, retaining some defective crystal lattices, resulting in a decrease in long-range order and crystallinity.

After the transformation of crystal form, it does not necessarily cause a decrease in solubility. The effect of crystal form transformation on solubility depends on the lattice energy of the new crystal form [37–39]. However, the amorphization of the crystal usually leads to a significant increase in solubility.

According to relevant reports, the amorphous state of a substance has a higher solubility than its crystalline state, and the core lies in the weakening of lattice binding caused by the decrease in lattice order, the increase in interfacial energy, and the optimization of solvation pathways [40–42]. Molecules in crystals are arranged in a highly ordered three-dimensional network, and lattice energy (such as intermolecular hydrogen bonds and van der Waals forces) must be overcome before dissolution. The amorphous region lacks long-range order, significantly weakens intermolecular forces, reduces the energy barrier required for dissolution, and increases thermodynamic solubility accordingly [43–45]. In addition, the molecular conformation in amorphous form is more flexible, polar groups (such as hydroxyl and carbonyl groups) are fully exposed, and hydrogen bonding interactions with water molecules are initiated in advance, resulting in a decrease in activation entropy for the formation of solvation shells, thus exhibiting higher equilibrium solubility at the macroscopic level [46,47]. Based on the above analysis, the partially amorphous MSC-puerarin has higher solubility and dissolution.

### Fourier transform infrared spectroscopy (FTIR) analysis

**Fig 7:**
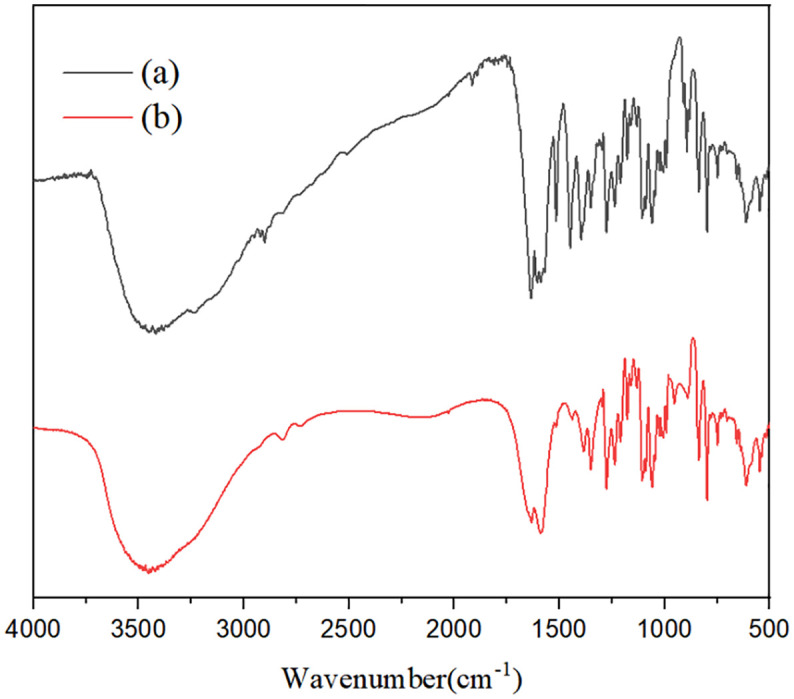
FTIR of puerarin. **a,** FTIR of raw puerarin. **b,** FTIR of MSC-puerarin. The shifted peaks indicate rearrangement of hydrogen bonding and π-conjugation environments.

**Fig 8:**
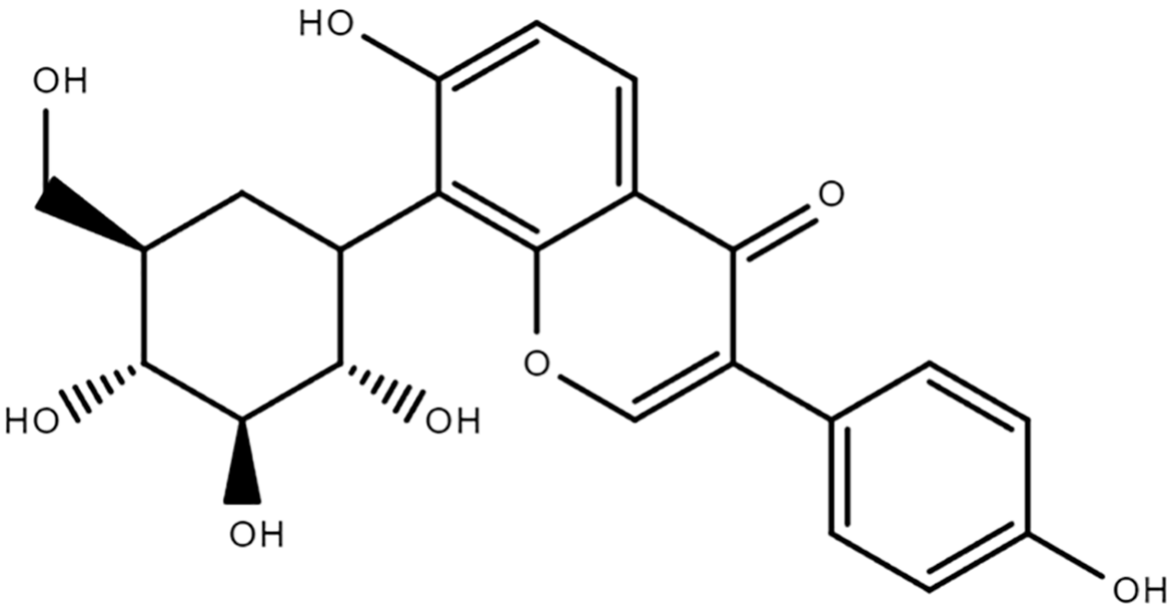
Chemical structure of puerarin.

The experiment used FTIR to characterize the raw material of puerarin and the MSC-puerarin, as shown in Fig 7. The raw material exhibited a wide and strong absorption peak in the range of 2500−3700 cm^-1^. Combined with the chemical structure formula of puerarin (Fig 8), it can be inferred that this peak corresponds to O-H stretching vibration, formed by hydrogen bonding between the hydroxyl group on the carbon chain and the aromatic ring; The strong peak presented in the range of 1550−1750 cm^-1^ corresponds to the C = O stretching vibration of the conjugated ketone carbonyl in the benzo-γ-pyrone moiety of the isoflavone skeleton, characteristic of the core structure of puerarin; Multiple moderate intensity absorption peaks appear at 1450−1550 cm^-1^, corresponding to the stretching vibration of the conjugated double bond of the benzene ring; The characteristic peak corresponding to the C-H bending vibration (methylene) on the carbon chain of puerarin structure appears in the range of 1350−1480 cm^-1^; After MSC treatment, the peak positions of the main functional groups are basically retained, but there are changes in peak shape and peak position displacement; The strong peak corresponding to the C = O stretching vibration in the range of 1550−1750 cm^-1^ has undergone a change in peak shape, and there is a slight shift in the peak positions of the two peaks. The peak positions of the two peaks shift from 1631 cm^-1^ and 1584 cm^-1^ to 1633 cm^-1^ and 1595 cm^-1^, and the absorption peak in the range of 2500−3700 cm^-1^ also shows a certain degree of peak shape change. Both indicate that the external environment of carbonyl and hydroxyl groups has changed. Considering that carbonyl and hydroxyl groups are the main hydrogen bonding groups, the structure of puerarin may undergo recombination in a hydrogen bonding environment; The peaks at 1450–1550 cm^-1^ exhibit peak shifts ranging from 5−20 cm^-1^, indicating a change in the conjugated environment of the phenyl group in the structure of puerarin. Combined with the structure of puerarin, it can be inferred that the original π... π conjugation of the phenyl group may have formed a new conjugated structure with carboxylic acid groups or C-H bonds. By combining the results of infrared spectroscopy with powder X-ray diffraction analysis, it can be concluded that the changes in peak shape and position may be due to partial amorphization caused by crystal defects. Partial amorphization leads to changes in the external environment of the main functional groups such as carbonyl, hydroxyl, and phenyl groups in the structure of puerarin. Non covalent bonding forces such as hydrogen bonds and π bonds are rearranged, which in turn affects the infrared spectrum peaks of the substance.

Similarly, the results of FTIR also confirmed the analysis of partial amorphization of puerarin in powder X-ray diffraction experiments. In fact, the MSC-puerarin did not show the disappearance of old absorption peaks or the formation of new absorption peaks in FTIR analysis. Therefore, the MSC treatment did not cause damage to the molecular structure of puerarin, and only induced changes in the lattice microenvironment through physical effects.

### Thermogravimetric analysis (TGA)

**Fig 9:**
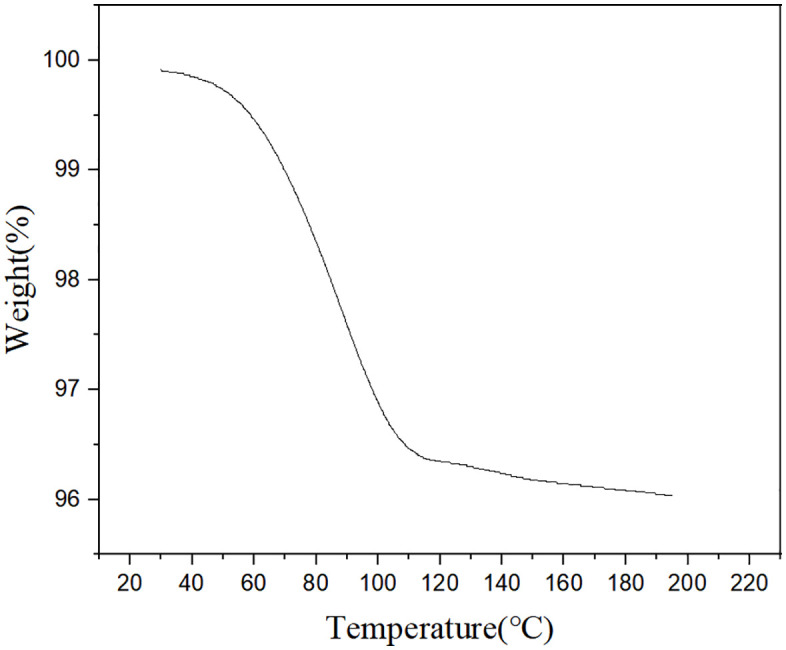
TGA of MSC-puerarin.

In order to clarify the properties of the broad thermal events observed at approximately 50 ° C in the DSC spectra of MSC-puerarin, TGA was applied for thermal analysis of MSC-puerarin (Fig 9). TGA of MSC-puerarin revealed a two-stage mass loss totaling approximately 4.0% between 30°C and 195°C, which is in excellent agreement with the theoretical value of 4.17% for puerarin monohydrate and the Karl Fischer measurement of 4.15%. The first-stage dehydration (30–120°C) accounted for approximately 3.6% mass loss, representing the primary dehydration event. Above 120°C, the mass change was minimal with no distinct step transition.

Correlation with the DSC thermogram demonstrated that the onset of TGA mass loss closely corresponds to the onset of the DSC endotherm, confirming that the thermal event at ~50°C is attributable to desolvation of the hydrated form. The broad thermal event observed at approximately 50°C in the DSC thermogram is therefore assigned to dehydration of the monohydrate. A glass transition of a minor amorphous fraction may exist but is likely masked by thermal overlap with the extensive dehydration endotherm. While DSC cannot definitively etct the glass transition process, the result of PXRD and FTIR independently confirms the presence of amorphous material. The absence of a detectable T_g_ does not negate the existence of amorphous regions; rather, it reflects the technical limitations of conventional DSC for this specific sample configuration, which represents a direction for our future efforts.

The current experimental results are sufficient to support our core conclusion that MSC-puerarin consists primarily of near-stoichiometric puerarin monohydrate crystals with a partial amount of amorphous material.

## Conclusion

This work is the first to apply MSC to puerarin, significantly improving its solubility and dissolution rate. After MSC treatment, the particle size of puerarin significantly decreased, MSC-puerarin underwent a crystal transformation into puerarin monohydrate, with a decrease in crystallinity and partial amorphization. In summary, this work systematically studied the effects of MSC treatment on the size, crystal form and morphology, bonding information, and solubility and dissolution improvement of puerarin, and lays a foundation for the development of puerarin oral formulations.

## Supporting information

S1 Data Raw data. (XLSX)

## References

[1] Chaudhary A, Nagaich U, Agrawal DK. Enhancement of solubilization & bioavailability of poorly soluble drug. 2012.

[2] Murdande SB, Pikal MJ, Shanker RM, Bogner RH. Aqueous solubility of crystalline and amorphous drugs: challenges in measurement. Pharm Dev Technol. 2011;16(3):187–200. doi: 10.3109/10837451003774377 20429826

[3] Cho H-H, Park J-W, Liu CCK. Effect of molecular structures on the solubility enhancement of hydrophobic organic compounds by environmental amphiphiles. Environ Toxicol Chem. 2002;21(5):999–1003. doi: 10.1897/1551-5028(2002)021<0999:eomsot>2.0.co;2 12013147

[4] Hong M, Wu N, Wang Q, Zhou R, Zhu B, Qi M-H, et al. Insight into the relationship of aqueous solubility and single-crystal structure of Harmine’s new organic salt forms. Cryst Growth Des. 2024;24(8):3114–32. doi: 10.1021/acs.cgd.3c00891

[5] Desai J, Patel S, Manjappa A, Patel A, Patel T. Investigation of solid lipid nanoparticles as oral delivery of Paclitaxel for enhanced absorption. J Res Pharm. 2024;28.

[6] Li J, Leung SSY, Chan EHY, Jiang C, Ho ETY, Zuo Z. Significantly increased aqueous solubility of piperine via nanoparticle formulation serves as the most critical factor for its brain uptake enhancement. Int J Nanomedicine. 2025;20:3945–59. doi: 10.2147/IJN.S506827 40181831 PMC11967364

[7] Pereira CN, Paim L, Cruz JAD, Agnol LD, Mauler RS, Bianchi O. Determination of carbon nanoparticle dispersion solubility parameters using the classic Hansen and the DiPEVa method. J Mol Liq. 2024;393:12.

[8] Pindelska E, Sarna A, Duszczyk M, Zep A, Madura ID. Enhancing febuxostat solubility through cocrystal formation: role of substrate selection and amide coformers. Int J Mol Sci. 2025;26(7):3004. doi: 10.3390/ijms26073004 40243639 PMC11988470

[9] Xu H, Tang Y, Wu Q, Li W, Zhou L, Wang M. A drug–drug cocrystal strategy to regulate stability and solubility: a case study of temozolomide/caffeic acid. J Mol Struct. 2024;1312.

[10] Nijhawan M, Sirisha N, Bhavana J, Neelima R, Aleti R, Sailaja G, et al. Formulation and evaluation of solid dispersion of felodipine by hot melt extrusion for enhancement of solubility. Asian J Pharm Res Health Care. 2024;16:178–83.

[11] Rashmi K, Ash K, Dev A. Optimization and assessment of three generation solid dispersion for enhancement of solubility and dissolution for montelukast sodium. Drug Dev Ind Pharm. 2025;51(5):409–18. doi: 10.1080/03639045.2025.2477722 40062530

[12] Paradkar A, Maheshwari M, Kamble R, Grimsey I, York P. Design and evaluation of celecoxib porous particles using melt sonocrystallization. Pharm Res. 2006;23(6):1395–400. doi: 10.1007/s11095-006-0020-4 16741659

[13] El‐Kamel AH. Improvement of physicochemical and biopharmaceutical properties of flurbiprofen using melt sonocrystallization technique. Drug Dev Res. 2008;69(1):34–41. doi: 10.1002/ddr.20225

[14] Jagtap VA, Vidyasagar G, Dvivedi SC. Solubility enhancement of rosiglitazone by using melt sonocrystallization technique. J Ultrasound. 2014;17(1):27–32. doi: 10.1007/s40477-014-0074-9 24616749 PMC3945191

[15] Manish M, Harshal J, Anant P. Melt sonocrystallization of ibuprofen: effect on crystal properties. Eur J Pharm Sci. 2005;25(1):41–8. doi: 10.1016/j.ejps.2005.01.013 15854799

[16] Zhen Z, Lam T-N, et al. Radix Puerariae: an overview of its chemistry, pharmacology, pharmacokinetics, and clinical use. J Clin Pharmacol. 2013. 10.1002/jcph.96 23677886

[17] Guerra MC, Speroni E, Broccoli M, Cangini M, Pasini P, Minghett A, et al. Comparison between Chinese medical herb Pueraria lobata crude extract and its main isoflavone puerarin antioxidant properties and effects on rat liver CYP-catalysed drug metabolism. Life Sci. 2000;67(24):2997–3006. doi: 10.1016/s0024-3205(00)00885-7 11133012

[18] Wu Y, Wang X, Fan E. Optimisation of ultrasound-assisted extraction of puerarin and total isoflavones from Puerariae Lobatae Radix (Pueraria lobata (Wild.) Ohwi) with response surface methodology. Phytochem Anal. 2012;23(5):513–9. doi: 10.1002/pca.2349 22259187

[19] Kintzios S, Makri O, Pistola E, Matakiadis T, Shi HP, Economou A. Scale-up production of puerarin from hairy roots of Pueraria phaseoloides in an airlift bioreactor. Biotechnol Lett. 2004;26(13):1057–9. doi: 10.1023/B:BILE.0000032963.41208.e8 15218379

[20] Zhou Y-X, Zhang H, Peng C. Puerarin: a review of pharmacological effects. Phytother Res. 2014;28(7):961–75. doi: 10.1002/ptr.5083 24339367

[21] Li D, Hu Y, Yang K. Protective effect of puerarin on endothelial dysfunction of heat shock protein 60 induced specific immunity in apolipoprotein E-null mice. Zhongguo Zhong Xi Yi Jie He Za Zhi. 2006;26 Suppl:4–6. 17569333

[22] Skibsted LH. Puerarin as an antioxidant fluorescence probe. 2008.

[23] Han R-M, Tian Y-X, Becker EM, Andersen ML, Zhang J-P, Skibsted LH. Puerarin and conjugate bases as radical scavengers and antioxidants: molecular mechanism and synergism with beta-carotene. J Agric Food Chem. 2007;55(6):2384–91. doi: 10.1021/jf062796c 17300199

[24] Jin SE, Son YK, Min BS, Jung HA, Choi JS. Anti-inflammatory and antioxidant activities of constituents isolated from Pueraria lobata roots. Arch Pharm Res. 2012;35:823–37. 22644850 10.1007/s12272-012-0508-x

[25] Mahdy HM, Mohamed MR, Emam MA, Karim AM, Abdel-Naim AB, Khalifa AE. The anti-apoptotic and anti-inflammatory properties of puerarin attenuate 3-nitropropionic-acid induced neurotoxicity in rats. Can J Physiol Pharmacol. 2014;92(3):252–8. doi: 10.1139/cjpp-2013-0398 24593790

[26] Xu M-E, Xiao S-Z, Sun Y-H, Zheng X-X, Ou-Yang Y, Guan C. The study of anti-metabolic syndrome effect of puerarin in vitro. Life Sci. 2005;77(25):3183–96. doi: 10.1016/j.lfs.2005.03.036 16005472

[27] Yue HW, Hu XQ. Pharmacologic value of radix Puerariae and puerarine on cardiovascular system. Zhongguo Zhong Xi Yi Jie He Za Zhi. 1996;16(6):382–4. 9387770

[28] Deng Y, Esk NG, Yeung J, Kwan YW, Lau C, Zuo Z, et al. Cerebral vasodilator activities of major active constituents of a Danshen and Gegen formulation on rat basilar artery. 2011.

[29] Ma R, Zhao L, Zhao Y, Li Y. Puerarin action on stem cell proliferation, differentiation and apoptosis: therapeutic implications for geriatric diseases. Phytomedicine. 2022;96:153915. doi: 10.1016/j.phymed.2021.153915 35026503

[30] Wu C, Qi H, Chen W, Huang C, Su C, Li W, et al. Preparation and evaluation of a Carbopol/HPMC-based in situ gelling ophthalmic system for puerarin. Yakugaku Zasshi. 2007;127(1):183–91. doi: 10.1248/yakushi.127.183 17202799

[31] Yu A, Wang H, Wang J, Cao F, Gao Y, Cui J, et al. Formulation optimization and bioavailability after oral and nasal administration in rabbits of puerarin-loaded microemulsion. J Pharm Sci. 2011;100(3):933–41. doi: 10.1002/jps.22333 20862776

[32] Saraswat G. Experimental evaluation of puerarin nanoparticle with reference to its contragestative potential. 2016.

[33] Wang Z-X, Wang Y-Z, Chen X, Wu A-J, Liu W, Li H-J. Construction of chitosan hydrochloride-carboxymethyl chitosan nanoparticles using anti-solvent method for the co-delivery of puerarin and resveratrol. J Food Sci. 2025;90(1):e17628. doi: 10.1111/1750-3841.17628 39731710

[34] Pang Z, Wei Y, Wang N, Zhang J, Gao Y, Qian S. Gel formation of puerarin and mechanistic study during its cooling process. Int J Pharm. 2018;548(1):625–35. doi: 10.1016/j.ijpharm.2018.07.038 30016673

[35] Grujicic M, Saylor JR, Beasley DE, Derosset WS, Helfritch D. Computational analysis of the interfacial bonding between feed-powder particles and the substrate in the cold-gas dynamic-spray process. Appl Surf Sci. 2003;219:211–27.

[36] Ali S, Smaby JM, Brockman HL, Brown RE. Cholesterol’s interfacial interactions with galactosylceramides. Biochemistry. 1994;33(10):2900–6. doi: 10.1021/bi00176a020 8130203 PMC4067055

[37] Perlovich GL, Volkova TV, Bauer-Brandl A. Towards an understanding of the molecular mechanism of solvation of drug molecules: a thermodynamic approach by crystal lattice energy, sublimation, and solubility exemplified by paracetamol, acetanilide, and phenacetin. J Pharm Sci. 2006;95(10):2158–69. doi: 10.1002/jps.20674 16883556

[38] Salahinejad M, Le TC, Winkler DA. Aqueous solubility prediction: do crystal lattice interactions help? Mol Pharm. 2013;10(7):2757–66. doi: 10.1021/mp4001958 23718811

[39] Braun DE, Gelbrich T, Kahlenberg V, Tessadri R, Wieser J, Griesser UJ. Stability of solvates and packing systematics of nine crystal forms of the antipsychotic drug aripiprazole. Cryst Growth Des. 2008;9(2):1054–65. doi: 10.1021/cg8008909

[40] Hancock BC, Parks M. What is the true solubility advantage for amorphous pharmaceuticals? Pharm Res. 2000. 10.1023/a:1007516718048 10870982

[41] Marsac PJ, Li T, Taylor LS. Estimation of drug-polymer miscibility and solubility in amorphous solid dispersions using experimentally determined interaction parameters. Pharm Res. 2009;26(1):139–51. doi: 10.1007/s11095-008-9721-1 18779927

[42] Babu NJ, Nangia A. Solubility advantage of amorphous drugs and pharmaceutical cocrystals. Cryst Growth Des. 2011;11(7):2662–79. doi: 10.1021/cg200492w

[43] Murdande SB, Pikal MJ, Shanker RM, Bogner RH. Solubility advantage of amorphous pharmaceuticals: II. Application of quantitative thermodynamic relationships for prediction of solubility enhancement in structurally diverse insoluble pharmaceuticals. Pharm Res. 2010;27(12):2704–14. doi: 10.1007/s11095-010-0269-5 20859662

[44] Pan X, Julian T, Augsburger L. Increasing the dissolution rate of a low-solubility drug through a crystalline-amorphous transition: a case study with indomethacin [correction of indomethicin]. Drug Dev Ind Pharm. 2008;34(2):221–31. doi: 10.1080/03639040701580606 18302042

[45] Wojnarowska Z, Grzybowska K, Hawelek L, Dulski M, Wrzalik R, Gruszka I, et al. Molecular dynamics, physical stability and solubility advantage from amorphous indapamide drug. Mol Pharm. 2013;10(10):3612–27. doi: 10.1021/mp400116q 24070615

[46] Taylor LS, Zografi G. Sugar-polymer hydrogen bond interactions in lyophilized amorphous mixtures. J Pharm Sci. 1998;87(12):1615–21. doi: 10.1021/js9800174 10189276

[47] Zilberman M, Siegmann A, Narkis M. Structure and properties of 6/6.9 copolyamide series. I. Amorphous phase. J Appl Polym Sci. 1996;59:581–7.

